# Acceptability of Automated Robotic Clinical Breast Examination: Survey Study

**DOI:** 10.2196/42704

**Published:** 2023-04-03

**Authors:** George P Jenkinson, Natasha Houghton, Nejra van Zalk, Jo Waller, Fernando Bello, Antonia Tzemanaki

**Affiliations:** 1 Bristol Robotics Laboratory Department of Mechanical Engineering University of Bristol Bristol United Kingdom; 2 Centre for Engagement and Simulation Science Department of Surgery and Cancer Imperial College London London United Kingdom; 3 Dyson School of Design Engineering Imperial College London London United Kingdom; 4 Cancer Prevention Group School of Cancer & Pharmaceutical Sciences King’s College London London United Kingdom

**Keywords:** breast cancer detection, automated diagnosis, breast examination, health care robotics, patient and public involvement, participatory design, user acceptability, mammography, breast cancer

## Abstract

**Background:**

In the United Kingdom, women aged 50 to 70 years are invited to undergo mammography. However, 10% of invasive breast cancers occur in women aged ≤45 years, representing an unmet need for young women. Identifying a suitable screening modality for this population is challenging; mammography is insufficiently sensitive, whereas alternative diagnostic methods are invasive or costly. Robotic clinical breast examination (R-CBE)—using soft robotic technology and machine learning for fully automated clinical breast examination—is a theoretically promising screening modality with early prototypes under development. Understanding the perspectives of potential users and partnering with patients in the design process from the outset is essential for ensuring the patient-centered design and implementation of this technology.

**Objective:**

This study investigated the attitudes and perspectives of women regarding the use of soft robotics and intelligent systems in breast cancer screening. It aimed to determine whether such technology is theoretically acceptable to potential users and identify aspects of the technology and implementation system that are priorities for patients, allowing these to be integrated into technology design.

**Methods:**

This study used a mixed methods design. We conducted a 30-minute web-based survey with 155 women in the United Kingdom. The survey comprised an overview of the proposed concept followed by 5 open-ended questions and 17 closed questions. Respondents were recruited through a web-based survey linked to the Cancer Research United Kingdom patient involvement opportunities web page and distributed through research networks’ mailing lists. Qualitative data generated via the open-ended questions were analyzed using thematic analysis. Quantitative data were analyzed using 2-sample Kolmogorov-Smirnov tests, 1-tailed *t* tests, and Pearson coefficients.

**Results:**

Most respondents (143/155, 92.3%) indicated that they would definitely or probably use R-CBE, with 82.6% (128/155) willing to be examined for up to 15 minutes. The most popular location for R-CBE was at a primary care setting, whereas the most accepted method for receiving the results was an on-screen display (with an option to print information) immediately after the examination. Thematic analysis of free-text responses identified the following 7 themes: women perceive that R-CBE has the potential to address limitations in current screening services; R-CBE may facilitate increased user choice and autonomy; ethical motivations for supporting R-CBE development; accuracy (and users’ perceptions of accuracy) is essential; results management with clear communication is a priority for users; device usability is important; and integration with health services is key.

**Conclusions:**

There is a high potential for the acceptance of R-CBE in its target user group and a high concordance between user expectations and technological feasibility. Early patient participation in the design process allowed the authors to identify key development priorities for ensuring that this new technology meets the needs of users. Ongoing patient and public involvement at each development stage is essential.

## Introduction

### Background

Breast cancer is the leading cause of cancer mortality in women worldwide [1]. Almost 11,400 women a year died of breast cancer in the United Kingdom between 2015 and 2017 [2]. However, the mortality rate of breast cancer is falling, with a reduction from 60 per 100,000 in 1989 to 33 per 100,000 in 2017 [2,3]. This trend correlates with the introduction of widespread breast cancer screening using x-ray mammography [4]. In the United Kingdom, mammography is offered to women aged 50 to 70 years through the National Health Service (NHS) Breast Cancer Screening Programme [3]. Screening is estimated to reduce the relative risk of breast cancer mortality by 20% [5] and is linked to many lives saved each year [6].

However, mammography is not suitable for all groups who could benefit from breast cancer screening [7]. For example, 10% of invasive breast cancers occur in younger women (aged <45 years) in the United Kingdom at a rate of 235 per 100,000 [8], a group for whom mammography is not recommended because of its considerably decreased sensitivity in dense breast tissue [7]. This is particularly concerning as young women diagnosed with breast cancer are at higher risk of developing aggressive subtypes and have a poorer prognosis [9]. Mammography is also inappropriate for pregnant women as the low-dose radiation used poses a potential risk during lactation [9]. Furthermore, some women may be unable to tolerate mammography because of pain or discomfort [10]. A breast cancer screening modality that extends services to these groups has the potential to save years of life [11]. Identifying a screening alternative has been challenging. The most effective means of diagnosis (eg, triple assessment and magnetic resonance imaging) are often invasive or costly and unfeasible as screening modalities [12].

A promising alternative is clinical breast examination (CBE) [13,14]. A recent randomized controlled trial of CBE breast cancer screening involving >150,000 women in India demonstrated a 15% reduction in breast cancer–related mortality and a 10% relative risk reduction in the diagnosis of stage-III or stage-IV disease [15]. An overview of systematic reviews assessing the effectiveness of CBE screening identified indirect evidence that CBE has the same effect as mammography when performed well [16]. However, there are several challenges to ensuring that CBE is consistently “well performed.” CBE screening effectiveness may be affected by variations in examination proficiency, training of health care professionals (HCPs), and a lack of standard documentation [17-21].

Recent advances in technology may provide a solution to these challenges. Robotics-assisted procedures have expanded rapidly in recent decades [22,23], and existing literature suggests that health users are increasingly more accepting of artificial intelligence (AI) and machine learning algorithms in cancer screening [24-26]. It is theoretically feasible to create a fully automated robotic CBE (R-CBE) platform by combining soft robotic technology and machine learning algorithms trained by breast specialists. This could offer much-needed standardization of CBE. R-CBE also has the potential to extend screening services to currently underserved groups as it is not reliant on radiation or affected considerably by tissue density. As health policy makers are discussing a risk-stratification approach to breast cancer screening, the cheap and low-risk modality of R-CBE may find further use as part of a strategy to classify the personalized risk level of an individual by measuring physiological properties such as mammographic density [27,28].

**Figure 1:**
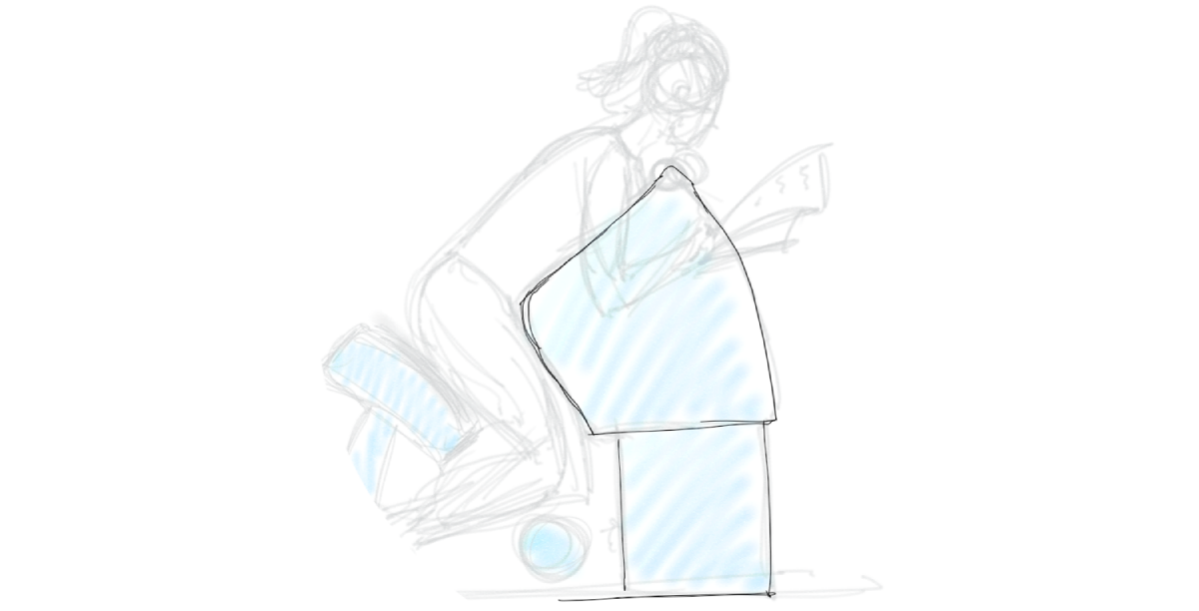
Automated Robotic Examination Intelligent System user interface (conceptual diagram).

The Automated Robotic Examination Intelligent System (ARTEMIS), a novel robotic system for automated CBE, is currently being developed by our research team with support from Cancer Research United Kingdom (CRUK). ARTEMIS aims to combine soft robotic technology with a machine learning platform to allow for fully automated CBE and interpretation of results. The platform could be used by women without direct clinical supervision (Figure 1). A prototype is currently in the early stages of development [29-31]. Although such a platform may be capable of effectively performing and interpreting CBE, the voices of potential users are essential in determining how this should be designed and implemented. Creating technology and a service that is acceptable to end users (and meets their needs) will be crucial in determining the uptake of this type of technology.

Very little published literature is available on the acceptability of intelligent systems that interface directly and independently with users. We identified only 1 study assessing the acceptability of autonomous robotic systems that interface directly with users in health care. This study used robotics to perform basic patient assessment tasks (eg, measuring vital signs and inserting intravenous catheters) and concluded that this would be acceptable [32]. We did not identify any publications exploring the acceptability of intelligent robotic services that directly interact with users in cancer screening or diagnostics.

### Objectives

This study investigated whether R-CBE is theoretically acceptable to potential users and explored the attitudes, perspectives, and concerns of women regarding the use of intelligent robotic technology in breast cancer screening. It identified key factors that determine whether (and how) the technology would meet the needs of patients, allowing these to be integrated into the prototype design. We adopted the definition of acceptability proposed by Sekhon et al [33]: “a multi-faceted construct that reflects the extent to which people receiving a healthcare intervention consider it to be appropriate, based on anticipated or experienced cognitive and emotional responses to the intervention.” We conducted a web-based survey of 155 women in the United Kingdom to investigate the following questions: (1) Is there a perceived need for R-CBE? (2) What elements of the R-CBE user interface are most important to women? (3) Is this technology likely to be acceptable to potential users? To the best of our knowledge, this is the first study assessing the acceptability of a fully automated and intelligent patient examination system that interacts directly with users for breast cancer screening.

## Methods

A mixed methods approach was used to collect and analyze qualitative and quantitative data from the survey.

### Survey Development

The survey consisted of 5 open-ended questions and 17 closed questions with a separate free-text section for respondents to share additional information. A brief overview of the proposed ARTEMIS concept was provided to respondents (Multimedia Appendix 1). This included Figure 1 and a description of how the user might interact with the palpation platform but had no technical details or any information on the accuracy of the device. Our aim was to allow the respondents to freely think about factors that might affect their use of the hypothetical service without imposing any of our priors.

Key constructs were identified based on a review of the health intervention acceptability and health technology literature, our broader knowledge of health technology, and support from the CRUK patient and public involvement specialist team and the London In Vitro Diagnostics Co-operative. We did not identify a fully validated model suitable for our research questions; instead, 2 frameworks were combined with questions selected to cover essential constructs from both. The first was the Theoretical Framework of Acceptability for health care interventions proposed by Sekhon et al [33]. The second was the Unified Theory of Acceptance and Use of Technology developed by Venkatesh et al [34], which has been widely used in research exploring the acceptance of ITs [35]. The resultant key constructs encompassed affective attitudes, perceived effectiveness, ethicality, self-efficacy, effort expectancy, social influence, and facilitating conditions.

The questions were carefully designed to illuminate implicit assumptions and ensure that all key constructs were considered while maintaining an accessible and nonleading language [36]. After a multistage drafting process, the survey was collated and tested on close contacts and members of the associated research department for appropriateness, readability, and ease of use to produce a final draft (Multimedia Appendix 1).

Closed questions allowed us to quantify the overall level of acceptability and desirability of specific features of the service (eg, interface, timing, and preferred location). Thematic analysis of qualitative data provided insights into the quantitative findings. This added richness to our understanding of potential users’ perspectives and attitudes toward the proposed ARTEMIS R-CBE and allowed us to build a more complete picture of acceptability.

### Recruitment and Data Collection

Female respondents aged between 20 and 70 years were recruited through a web-based survey linked to the CRUK patient involvement opportunities web page and newsletter and the People in Health West of England and Imperial Human Behaviour and Experience network mailing lists. This nonprobability, voluntary response sampling strategy was chosen because of its quick recruitment rate and ability to serve the exploratory nature of the study. With no hypothesis to test, the aim of the survey was to develop an initial understanding of the needs of the population, and so the bias introduced by self-selection was considered acceptable.

The 15-minute web-based survey was hosted on Qualtrics (Qualtrics International Inc), and 2 attention-check questions were added to ensure that respondents read each question carefully and also to exclude nonhuman (automated) respondents; this resulted in the expulsion of 1 set of responses because the attention questions were answered incorrectly. A further 15 questionnaires were discarded because they were incomplete, including incomplete attention questions, and 3 were discarded because they did not meet the inclusion criteria. This meant that, of 174 responses initiated, 155 (89.1%) completed the survey over 6 weeks between August 2020 and September 2020. Summing the size of each of the mailing lists gives a response rate of 9.26% (155/1674).

### Data Analysis

Quantitative analysis was conducted in MATLAB (MathWorks), and differences between groups based on demographics were identified using a 2-sample Kolmogorov-Smirnov test, explored using 1-tailed *t* tests, and reported where significant (full results available in the data set referenced in the Data Availability section). Pearson correlations were calculated where appropriate to quantify the strength of the associations. CIs were calculated for ranked questions assuming that the preferences were equidistant (1>2>3...).

Qualitative data were analyzed using a method designed around thematic analysis [36]. This allows for detailed exploration of patterns across a data set using a latent approach, with researchers gaining a rich understanding of respondents’ perspectives [36].

Themes were identified after familiarization with the open-text responses. To this end, 2 researchers independently identified a set of key themes within the responses, chosen with relevance to identifying the factors that influenced the respondents’ acceptance of the hypothetical technology. After combining these sets of themes, a single researcher divided each theme into concepts that tightly grouped responses within each theme. Salient ideas from these grouped concepts were then extracted to describe the outcomes of the responses as a whole.

The raw data are available from the source provided in the Data Availability section at the end of this paper.

### Ethics Approval

The study received ethics approval from the Imperial College Research Ethics Committee (20IC6129).

## Results

### Quantitative Results

#### Demographics

The average age of the respondents was 49.8 (SD 12.7; range 21-70) years. “White” ethnic (142/155, 91.6%) and university-educated (119/155, 76.8%) backgrounds were overrepresented among survey respondents. Our study population also had a higher incidence of personal history of breast cancer (28/155, 18.1%) compared with the general adult population (4.46% [8]). Respondents were overwhelmingly in favor of screening programs (143/155, 92.3%) and the use of technology in health care (146/155, 94.2%). The demographic data are summarized in Table 1, and attitudes toward screening and technology in health care in general are summarized in Table 2.

**Table 1:** Respondent demographics (N=155).

Demographics			Values, n (%)
**Age (years)**			
	21-44	54 (34.8)	
	45-59	48 (31)	
	60-70	51 (32.9)	
	Did not complete	2 (1.3)	
**Ethnicity**			
	White British	121 (78.1)	
	Other White	21 (13.5)	
	Black African	4 (2.6)	
	Indian	1 (0.6)	
	White and Black African	1 (0.6)	
	Pakistani	1 (0.6)	
	White and Black Caribbean	1 (0.6)	
	Chinese	1 (0.6)	
	Prefer not to say	3 (1.9)	
**Highest qualification**			
	Bachelor’s degree or higher	119 (76.8)	
	Vocational qualification (ONC^a^, BTEC^b^, or NVQ^c^)	13 (8.4)	
	A-Levels (or equivalent)	13 (8.4)	
	GCSE^d^ or O-Levels (or equivalent)	10 (6.5)	
**History of diagnosis of cancer**			
	Any (including breast cancer)	42 (27.1)	
	Breast cancer	28 (18.1)	

^a^ONC: Ordinary National Certificate.

^b^BTEC: Business and Technology Education Council qualification.

^c^NVQ: National Vocational Qualifications.

^d^GCSE: General Certificate of Secondary Education.

**Table 2:** Respondents’ attitudes toward breast cancer screening and technology in health care (N=155).

Questions and responses			Values, n (%)
**Do you think routine cancer screening tests are a good idea?**			
	Yes	143 (92.3)	
	No	4 (2.6)	
	Don’t know	8 (5.2)	
**What do you think of increased use of new technology in health care?**			
	Very bad idea	0 (0)	
	Bad idea	5 (3.2)	
	Good idea	48 (31)	
	Very good idea	98 (63.2)	
	Don’t know	5 (3.2)	

#### Overall Opinion Toward the Device

**Figure 2:**
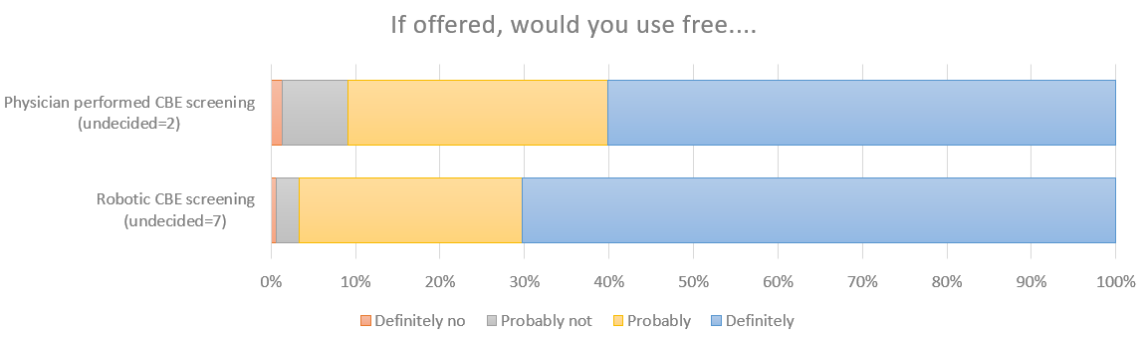
Overall opinion of the device. This demonstrates that the idea of a robotic system appeals to some respondents more so than the status quo. CBE: clinical breast examination.

Provided the R-CBE was as good as an HCP, 92.3% (143/155) of respondents said that they would either “definitely” (104/155, 67.1%) or “probably” (39/155, 25.2%) use an R-CBE service if it were offered. In comparison, 89.7% (139/155) of respondents said that they would “definitely” (92/155, 59.4%) or “probably” (47/155, 30.3%) use a service offering CBE by a trained HCP (Figure 2). This indicates that the answers to the 2 questions were similar, with a slight preference for R-CBE (2-sample Kolmogorov-Smirnov test; *P*=.40). Willingness to use an R-CBE service was moderately correlated with respondents’ likelihood of using new technology in general (*r*_155_=0.4014; *P*<.001).

Respondents were asked to indicate which factors would make them more likely to use R-CBE. The most popular option was receiving a “faster referral to specialist breast services” if required (144/155, 92.9% of respondents selected this option) and being able to drop in and use the device without an appointment (108/155, 69.7% of respondents). Other factors that influenced anticipated use are shown in Table 3.

**Table 3:** Factors to improve uptake, which provides insights into the respondents’ understanding of how a robotic system might best be of benefit to them (N=155).

What would make you more likely to use R-CBE^a^	Values, n (%)
Faster referral to a specialist	93 (60)
Drop-in appointments	70 (45.2)
Knowing what to expect before the appointment	68 (43.9)
My GP^b^ seeing the results	61 (39.4)
Confidential results	59 (38.1)
More technical information	39 (25.2)
Information on data protection	36 (23.2)

^a^R-CBE: robotic clinical breast examination.

^b^GP: general practitioner.

#### Device Features

The respondents favored the use of soft (rather than hard) robotic parts for the aspects of the device that would be in contact with their skin. Device features considered to be of most importance were availability of information on access to support from an HCP, appointment availability, cleanliness, and regular updates on examination progress throughout the procedure. The results are presented in Table 4.

A comparative analysis of the age groups revealed 3 significant differences. Each respondent scored a selection of features on a scale of 1 to 5. The age group of >60 years (the oldest) considered ease of appointment availability to be less important compared with the 2 younger age groups (>60 years vs 45 to 59 years: *mean difference [MD]*=0.37 and *P*=.02; >60 years vs <45 years: *MD*=0.43 and *P*=.04). Conversely, the age group of <45 years considered it less important to be able to adjust the speed of the device (<45 years vs 45 to 59 years: *MD*=0.59 and *P*=.04; <45 years vs >60 years: *MD*=0.87 and *P*=.001) or for the device to have disposable parts (<45 years vs 45 to 59 years: *MD*=0.89 and *P*=.002; <45 years vs >60 years: *MD*=0.70 and *P*=.02).

**Table 4:** Relative importance of device features. “On a scale from 1 (not at all important) to 5 (essential), how important is it that...”

Feature	Score, mean (95% CI)
The device provides links to support from HCPs^a^	4.26 (4.12-4.39)
Appointments are easily available	4.16 (4.04-4.28)
Information about the cleaning of the booth is available	4.10 (3.96-4.25)
The examination provides constant updates	4.08 (3.96-4.21)
The device is close to home or work	3.63 (3.49-3.78)
Parts of the device that are in contact with the skin are disposable	3.34 (3.14-3.55)
I am able to adjust the speed of the device’s parts that are in contact with the skin	2.76 (2.58-2.94)

^a^HCP: health care professional.

#### Location

Most respondents (130/155, 83.9%) preferred the booth to be located at a site associated with health care. The most popular location was at a general practitioner surgery, which generally provides point-of-contact care and triage between patients and specialist health services in the United Kingdom, followed by “inside a pharmacy.” Options not associated with health care (such as at a shopping center or in the workplace) were less popular. This difference was statistically significant. Location preference is shown in Table 5. The age group of >60 years favored the shopping center more compared with the other age groups (>60 years vs 45 to 59 years: *MD*=0.50 and *P*=.02; >60 years vs <45 years: *MD*=0.57 and *P*=.008).

**Table 5:** Location preference.

Rank	Option	Rank, mean (95% CI)
1	GP^a^ surgery	1.41 (1.23-1.50)
2	Pharmacy	2.15 (1.97-2.20)
3	Shopping center	3.77 (3.47-3.82)
4	Work	3.84 (3.52-3.91)

^a^GP: general practitioner.

#### Length of Examination

**Figure 3:**
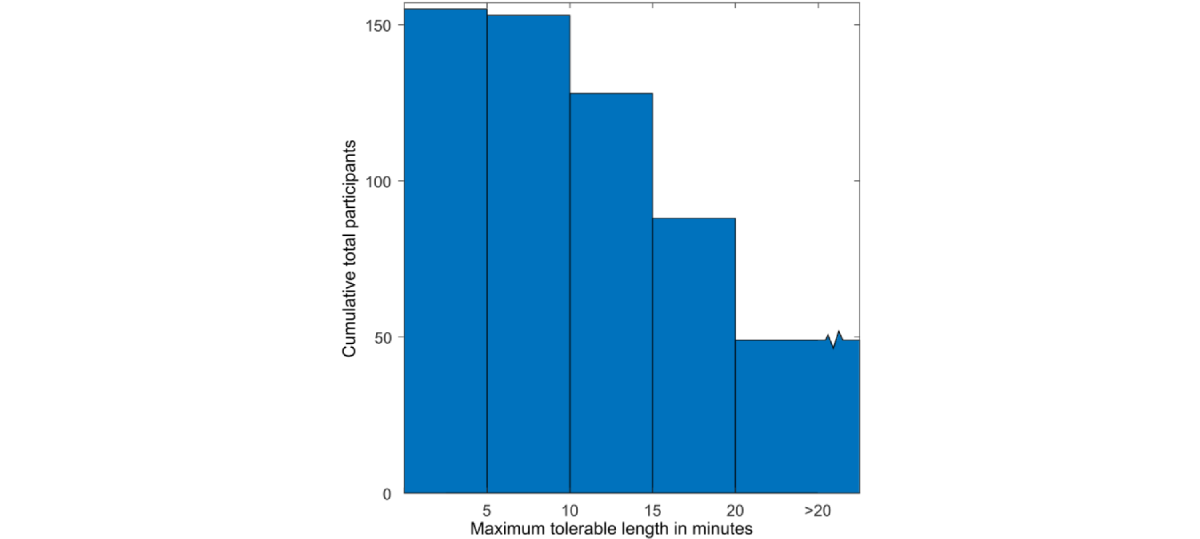
Cumulative tolerable examination duration. Nearly all respondents (153/155, 98.7%) were happy with an examination lasting up to 10 minutes, with a substantial minority (88/155, 56.8%) happy with a duration of up to 20 minutes.

**Figure 4:**
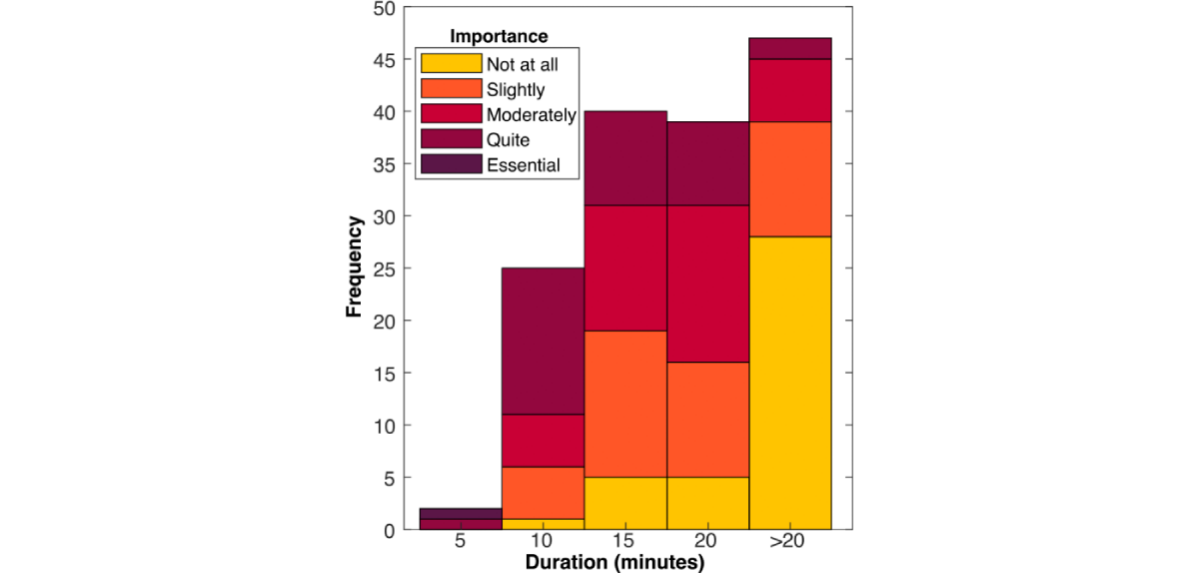
Tolerable length of examination. The respondents who preferred a shorter examination also considered duration a more important factor.

Most respondents (153/155, 98.7%) were willing to be examined for up to 10 minutes, 82.6% (128/155) were willing to be examined for up to 15 minutes, and 56.8% (88/155) were willing to be examined for 20 minutes. Interestingly, only 22.6% (35/155) of the respondents considered the time taken to carry out the examination to be “Quite important” (34/155, 21.9%) or “Essential” (1/155, 0.6%). When asked to rate “how important is it that the examination does not take longer [than the duration the respondent indicated]” on a scale of 0 (not at all important) to 5 (essential), the mean rating was 1.46. However, respondents who preferred a shorter examination duration were statistically more likely to report that it was important that the examination last no longer than they had indicated (*r*_153_=0.53; *P*<.001). These results are shown in Figures 3 and 4.

#### Communication of Results

Most users (128/155, 82.6%) preferred to receive information directly from the device, either displayed on the screen with a printout (mean rank 2.26) or received via email (mean rank 2.47). These options were statistically significantly more popular than the results being emailed to their physician first. This was true both in the case of a *normal* (mean rank 5.02) and an *abnormal* (mean rank 3.72) result. In the event of an *abnormal* result, the option “email to my doctor first” increased in preference (from sixth to fourth in the average rank) but remained comparatively unpopular. The most popular option for results communication was through a combination of written information and pictures (mean rank 1.62). Respondents without a university diploma or equivalent ranked seeing their results on-screen without a printout significantly higher than those with a university diploma or equivalent (healthy: *MD*=0.57 and *P*=.08; *abnormal*: *MD*=0.89 and *P*=.02). This suggests that the level of education may be an important discriminant when considering how results are communicated. Respondents highly valued the inclusion of information on appropriate follow-up and alternative explanations for identified *abnormalities*. The results are summarized in Tables 6-8.

It is worth noting that 6.5% (10/155) of respondents used the open-text “other” option to indicate that they would want to receive results from an HCP and not from the R-CBE device itself. All respondents (155/155, 100%) ranked this as their number 1 preference.

**Table 6:** Information receipt preferences.

Option	No referral advised, mean rank (95% CI)	Referral advised, mean rank (95% CI)	Rank change
Immediately on-screen+printout	2.26 (2.02-2.50)	2.41 (2.20-2.62)	−0.15
Emailed later	2.47 (2.26-2.68)	2.66 (2.49-2.83)	−0.19
Immediately on-screen	2.76 (2.57-2.96)	3.30 (3.06-3.53)	−0.52
SMS text message	4.10 (3.90-4.29)	4.61 (4.37-4.75)	−0.46
Posted later	4.24 (4.07-4.41)	4.25 (4.05-4.44)	−0.01
Emailed to physician first	5.02 (4.82-5.21)	3.72 (3.43-4.00)	+1.31
Other	7.40 (7.11-7.68)	6.40 (6.16-6.63)	0

**Table 7:** Information display preferences.

Rank	Option	Rank, mean (95% CI)
1	Written and pictures	1.62 (1.48-1.76)
2	Interactive app	2.02 (1.84-2.20)
3	Written only	2.53 (2.38-2.68)
4	Verbal summary	3.785 (3.6-3.97)

**Table 8:** Information content preferences (N=155).

What information would you like included in your results	Respondents, n (%)
Details on follow-up when referral is recommended	153 (98.7)
How to book a future R-CBE^a^ appointment	151 (97.4)
Other causes for an “abnormal” finding	122 (78.7)
Links to emotional support	108 (69.7)

^a^R-CBE: robotic clinical breast examination.

### Qualitative Results

#### Overview

Qualitative analysis of the free-text responses identified the following seven superordinate themes with respect to R-CBE: (1) women perceived that R-CBE has the potential to address limitations in current screening services, (2) R-CBE may facilitate increased user choice and autonomy, (3) ethical motivations for supporting R-CBE development, (4) accuracy (and users’ perceptions of accuracy) is a priority, (5) results management with clear communication is a priority for users, (6) integration with health services is key, and (7) device usability is important. These themes are summarized in the following sections. Quotes from the respondents illustrating the themes are shown in Table 9.

**Table 9:** Themes from thematic analysis with supporting quotes.

Theme and concept		Quotes
**R-CBE^a^ has the potential to address limitations in current services**		
	Provides reassurance	“I worry about my breast health. It would be reassuring to be able to check for irregularities.” [Respondent 090] “...to be able to regularly monitor for something like breast cancer would give me peace of mind.” [Respondent 008]
	Reluctance to “waste” physicians’ time	“I find [breast examination] difficult to do myself and don’t like to take up doctors time very often.” [Respondent 032] “I would also like regular check-ups and understand GPs need to prioritise other appointments.” [Respondent 097]
	Negative experiences with mammography	“When I have a mammogram it really hurts me. I often say that the machine is like torture.” [Respondent 129] “I would welcome any solution that is pain free.” [Respondent 129]
	Embarrassment during clinical examination	“Every time I see breast screening on TV there is a picture of women with a completely naked top half. This makes me feel very uncomfortable and puts me off screening.” [Respondent 053] “I would rather have a machine examine my breasts than a doctor. It would eliminate the feeling of embarrassment.” [Respondent 036] “...lack of human contact may encourage more women to use it.” [Respondent 087]
	Anxiety associated with awaiting results	“Every time I have a mammogram, I panic for 2-3 weeks waiting for the results.” [Respondent 134] “If results are available immediately then that’s better than waiting for test results and stops stress and anxiety.” [Respondent 106] “The possibility of having instant results is amazing.” [Respondent 142]
**R-CBE may facilitate increased user choice and autonomy**		
	Choice over appointment time, frequency, and location	“...it’s is [sic] more convenient if you have a bigger choice over appointment times.” [Respondent 039] “Freedom to choose when to use the device.” [Respondent 029] “...hopefully accessibility (location/appointments) are easier than going to the GP.” [Respondent 049] “...this would allow more frequent checks.” [Respondent 147]
	Increased sense of autonomy	“...the opportunity to be firmly in control of ones [sic] own health concerns is appealing.” [Respondent 143] “I believe the autonomy of this device may encourage more people to come forth for screening.” [Respondent 132]
**Ethical motivations for supporting R-CBE**		
	Support for population screening in general	“I like to spread the word about health screening, it’s very important to look after your health.” [Respondent 053] “I would support anything that encourages people to be tested.” [Respondent 054]
	Potential to increase access for underserved populations	“I think it is very important that women can have regular breast examinations that start at a younger age that [sic] mammograms!” [Respondent 007] “Digital Automation seems to be one way of improving life chances for black Cancer patients like myself.” [Respondent 144] “[a family member] has a learning difficulty, is deaf and is a wheelchair user. It has not been possible for her to have the benefit of regular breast screening. I am hopeful that this new device will help women like her in the future.” [Respondent 143]
	Reduced burden on the NHS^b^	“Technology is advancing and the population is growing. Using this technology in health care will help to free up our medical staff so that they can use their much needed skills working it [sic] areas where only human intervention is possible.” [Respondent 063] “It would seem to be an efficient screening tool that allows precious medically trained staff to do other jobs a machine cannot do.” [Respondent 111]
**Accuracy, and users’ perception of accuracy, is a priority**		
	R-CBE is only acceptable if users are convinced of its accuracy	“I think it is a very good idea...provided there is a definite level of assured accuracy.” [Respondent 062] “I would use it if I had confirmation that results are accurate.” [Respondent 054]
	Factors that increase confidence in accuracy	“...if the technology and device is proven through appropriate clinical studies.” [Respondent 155] “...how often it gets the diagnosis right, how often it gets it wrong.” [Respondent 022] “MHRA [Medicines and Health care products Regulatory Agency] approval.” [Respondent 041] “...some sort of checking procedure e.g. [sic] every 50th person is called in for manual checks.” [Respondent 054]
**Suitable results management with sensible communication**		
	Sensitivity concern about receiving results from R-CBE	“I worry about the emotional impact of an abnormal result being given via automated means.” [Respondent 006] “I think a human can usually be gentler with the feelings of patients.” [Respondent 022]
	Rapid results are preferred	“[I] wouldn’t want doctor involvement to delay my getting the result.” [Respondent 042] “I would much prefer the results at the time of the test.” [Respondent 134]
	Factors that optimize user experience when receiving results	“I’d want to know more about what an ‘abnormal’ result might mean—does it definitely mean cancer, or could it mean something else?” [Respondent 100] “If there is an abnormal result it will cause an amount of worry and anxiety and so any additional information that can be provided alongside the results such as emotional support and links to further information would be really useful.” [Respondent 142] “[if] the results are abnormal...an automatic urgent appointment should be made by the GP straight away.” [Respondent 079]
	Confidentiality and privacy are essential	“...all the physical privacy and data privacy issues [need to be] well thought through.” [Respondent 059] “To be screened in a booth, it would have to be entirely 100% privacy proof, confidential, and safe.” [Respondent 134]
**Integration with health services is key**		
	High trust placed in the NHS	“I would use a machine if it ran in tandem with NHS services.” [Respondent 022] “If it’s recommended by my GP or other relevant HCP.” [Respondent 141] “I would expect it to complement other services not replace them.” [Respondent 145]
	Geographic proximity to other health services	“I think I would feel more comfortable if the service was in a health care setting (e.g. GP/pharmacy), rather than in a more public space (e.g. work).” [Respondent 014] “I think the location should be somewhere linked to medical care/support—even if just near a first aider’s office.” [Respondent 146]
**Device usability is important**		
	Clear instructions required	“...if the instructions are fool-proof I think I could manage it.” [Respondent 063] “[needs] clear and understandable for everyone.” [Respondent 082] “A video demo would be helpful to maybe watch before attending.” [Respondent 148]
	Clear plan for managing technical difficulties	“I might need a little reassurance that the machine wasn’t going to run amok.” [Respondent 149] “My only reservation was if it went wrong and either used the wrong pressure or wouldn’t unclamp from the breast.” [Respondent 055] “Where to get help if the device didn’t work or stopped working during examination.” [Respondent 145] “...a panic or immediate stop function [with] the ability to cancel and walk away.” [Respondent 151]

^a^R-CBE: robotic clinical breast examination.

^b^NHS: National Health Service.

#### Women Perceive That R-CBE Has the Potential to Address Limitations in Current Screening Services

The limitations of current breast cancer screening services were raised frequently, and respondents perceived that R-CBE has the potential to address some of these limitations. “Check-ups” could provide regular reassurance lacking in current services. Respondents recognized that they could regularly self-examine (but lacked confidence to do so) or request regular examinations from a health practitioner (but did not want to waste the physician’s time). Pain associated with mammography was the most frequently cited limitation of breast cancer screening. Many respondents assumed that soft robotics would be more comfortable than a mammogram. R-CBE could also reduce the embarrassment of being seen unclothed by an HCP during mammography or CBE. Some respondents believed that a fully automated service that reduced this embarrassment was preferable to direct human involvement. Long waiting times to receive screening results were associated with anxiety, and the possibility of receiving rapid results from automated technology was highly appealing. This theme reflects the potential of R-CBE to address limitations in current services.

#### R-CBE May Facilitate Increased User Choice and Autonomy

R-CBE may be “more convenient” than other screening services, offering a wider choice of appointment times, location, and the frequency with which the service could be accessed. This increased choice over where and when, combined with the opportunity to complete screening without input from an HCP, was appealing and provided a sense of autonomy and control.

#### Ethical Motivations for Supporting R-CBE Development

Some respondents viewed R-CBE favorably on an ethical basis. For example, respondents suggested R-CBE (with the potential to be a convenient and accessible service) could increase screening among traditionally underserved populations such as young women, ethnic minorities, or people with disabilities. There was a desire to extend screening and cancer prevention on a population basis, irrespective of the modality, and strong support for the NHS. Respondents indicated that they would accept R-CBE if it reduced the burden on the NHS and HCPs. This reflects an underlying assumption that an automated device screening service would reduce the burden on the NHS. This assumption is explored further in the Discussion section. This theme indicates support for the R-CBE concept based on the respondents’ broader attitudes and ethical beliefs.

#### Accuracy, as well as Users’ Perception of Accuracy, Is a Priority

Acceptance of R-CBE was conditional, and respondents identified several factors required for R-CBE to be trustworthy. Chief among these was accuracy. Unsurprisingly, the requirement that the device have high levels of accuracy was mentioned by most respondents (132/155, 85.2%) unprompted. There was no clear required accuracy threshold. Some respondents wanted to see a service that was “as good as a mammogram,” others wanted to see a service “as good as a GP,” and others still “would use the device on the condition that it was better than a doctor.” However, there was a consensus that users should be provided with enough information to make their own informed decision as to whether R-CBE is accurate enough. Respondents suggested that users be given information on the sensitivity and specificity, ongoing monitoring of device performance, clinical trials completed, and regulatory approval to optimize trust. To be trustworthy, R-CBE must be highly accurate, and salient understandable information on how this accuracy is determined must be made available.

#### The Need for Suitable Results Management With Sensitive Communication

Communication of results in a sensitive manner was a key priority. Receiving screening results is anxiety-inducing, and the responses indicated that this is particularly true for technology-based services. Some respondents expressed concern about the ability of R-CBE to do this in a sufficiently sensitive manner. A small number of respondents felt that direct human involvement was essential in the event of an *abnormal* result. They felt strongly about this and described the idea of receiving an *abnormal* result from an automated device as “cold,” “impersonal,” and “abhorrent.” However, more respondents reported that rapid availability of results outweighed this disadvantage. Options for optimizing direct R-CBE results delivery were identified. These included ensuring an efficient follow-up process, providing information on possible causes of an *abnormal* result (options other than malignancy), and providing guidance on where users could access support if needed. It was also important to respondents that results management be private and confidential and that detailed information on data storage be available.

This theme illustrates the need for efficient, sensitive, private, and secure processes for managing results that place users first. Providing sufficient information to service users may optimize the experience and minimize the anxiety associated with receiving results.

#### Integration With Health Services Is Key

Along with timely follow-up of *abnormal* results, functional integration with the health service was highly valued. Adequate integration with the health service appeared to increase user confidence in the new technology. A high degree of trust was placed in the NHS, and integration with this would lend credibility to R-CBE. It was important that the new technology be an adjunct to existing services without reducing access to general practitioners or current NHS services. Geographic proximity to existing health services was also viewed positively as respondents perceived that this could improve integration and access to support. The trustworthiness of R-CBE appears to depend not only on the device itself but also on the extent to which it is integrated into the existing health system.

#### Device Usability Is Important

Acceptability was conditional on R-CBE being easy to use. People must also be confident that they can use the device without compromising accuracy. The importance of clear instructions was highlighted; providing a short instructional video was a suggested method of ensuring this. There was also a degree of anxiety regarding the possibility of malfunctions. Respondents wanted a clear procedure for dealing with technical difficulties. Suggestions for this included an emergency stop function and a process for calling for assistance. Clear instructions, a plan for malfunctions, and an emergency stop button would provide peace of mind and respect women’s autonomy by giving them control over the examination.

## Discussion

### Principal Findings

Responses were generally positive for a potential R-CBE service that is at least equivalent to a nonrobotic alternative. The overwhelming majority of respondents reported that they would use R-CBE screening if it were offered. Respondents recognized the potential of R-CBE to address an unmet need in current screening services by providing regular reassurance, reducing interpersonal embarrassment, reducing screening-associated pain, improving appointment availability, and offering rapid results. All these are barriers to screening uptake recognized in the existing literature [37].

The survey showed that a high level of sensitivity and specificity of this technology is an essential factor for acceptability. User acceptance in our survey was dependent on R-CBE being a highly accurate system.

The results of this study also complement the existing literature on AI diagnostics, which suggests that the public has a high level of trust in computerized decision-making in health care and that AI in cancer screening is increasingly accepted [24-26].

The acceptance of R-CBE was qualified. Our results complement the existing literature [38] by identifying high levels of trust as an essential property for the uptake of robotic and automated systems. Our data identified factors that are necessary for an R-CBE service to be considered trustworthy. Key among these are accuracy, usability, and communication. Respondents’ concerns regarding the lack of human connection, data privacy, and regulation of new health technology echoed similar concerns identified in a recent study exploring the public perception of AI mammography reading [26].

Our results indicate that most users are likely to accept autonomous screening if there is a well-established, efficient process for follow-up with a clinician if needed. This agrees with studies to date indicating that people are more accepting of intelligent systems working symbiotically with physicians or HCPs [24,39] but remain ambivalent about those that function independently [25].

This study provides important information to guide decision-making on R-CBE development, determine its viability as an investment, and inform our understanding of public attitudes toward intelligent health technology in cancer screening. Crucially, our results indicated a significant concordance between what is technically feasible and what is acceptable to users. For example, most respondents (128/155, 82.6%) were willing to be examined for up to 15 minutes and were also willing to receive results directly from ARTEMIS (in some format) rather than from an HCP. Research suggests that it is feasible to create an automated R-CBE service based on these acceptability characteristics [29-31].

### Limitations and Future Directions

From these results, we believe that R-CBE may offer a more patient-focused option that has the potential to increase screening uptake provided it can perform examinations with sufficient sensitivity and specificity.

To develop technologies seeking to provide the service of R-CBE or similar, these results provide appropriate targets to be met when evaluating their expected acceptability. For example, several respondents supported R-CBE because it would reduce the burden on the NHS and free up time for HCPs. Although early detection and intervention could reduce progression to advanced disease (and, therefore, reduce the treatment burden on the NHS), this assumption is only valid if R-CBE detects early disease and allows for early intervention without overdiagnosis or excessive referrals to primary or specialist services.

As an investigative survey, the sample size was comparatively small, and the skewed distribution of demographic groups within the sample means that it was insufficiently powered to detect nuanced differences between them. A larger sample size with a demographic distribution representative of the wider population would be needed to identify whether the subtle differences in preference between demographic groups in this study are statistically significant and externally valid in the general population.

The demographics of the respondents were also not representative of the UK population. First, Black and minority ethnic groups were underrepresented. The data may not accurately capture the needs, thoughts, attitudes, and perspectives of these demographics. This is of particular concern as these groups are at an elevated risk of breast cancer and face the greatest barriers to screening [40,41]. Reaching these groups in future research is essential as they may benefit substantially from widening screening. Achieving this is likely to require targeted methods.

In addition, over three-quarters of our sample (119/155, 76.8%) had a degree-level education. Jonmarker et al [25] found a significant association between level of education and level of trust in technology. This is reflected in the very high levels of trust in technology reported in our sample. This reduces the generalizability of our results, with survey respondents being more likely to find R-CBE acceptable than the general population. The non–probability sampling used in this study may also introduce selection bias—it is possible that women who had a history of engaging with existing breast cancer screening programs were more likely to answer the survey, which might have contributed to overestimation of the acceptability of R-CBE screening. The particular method of electronic survey requires respondents to have ready access to a compatible device connected to the internet and be literate at using it, inherently excluding those who do not fulfill both criteria.

The ARTEMIS R-CBE is currently in development (part of this system is described in the study by Jenkinson et al [31]); the responses relate to a theoretical service. Further research will be needed to establish the acceptability of the specific service among users as development continues, as well as an assessment of its cost and accuracy. Future research would benefit from a larger and more diverse sample size that better represents the population. Our team is currently undertaking further qualitative research via focus groups to better understand the requirements of trustworthy and acceptable R-CBE and automated breast cancer screening more generally. Despite the limitations outlined previously, the survey data allowed us to identify key priorities among potential users and provide valuable information for the research team. These findings may provide insights for others working in automated health technology development, particularly for cancer screening.

### Conclusions

R-CBE holds promise as a new modality of breast cancer screening. It could address limitations in current screening services, increase screening uptake, and provide a more patient-focused service. This investigative survey demonstrated that there is potential for high levels of acceptability of R-CBE among its target user group and a high concordance between user expectations and technological feasibility. However, the acceptability of R-CBE is conditional on users being confident that it is accurate, easy to use, able to communicate results sensitively, and well integrated with health services. These findings will contribute directly to prototype development and will be of interest to other researchers developing automated cancer screening and related health technologies. This study highlights the fact that the development of new technologies raises ethical and practical issues. The importance of public and patient involvement in health technology development to address these issues should not be underestimated. Patient and public involvement at each stage of development will be key to ensure that any future service meets the needs of the public.

## Appendix

Multimedia Appendix 1 Questionnaire.

## Abbreviations

AI: artificial intelligence
ARTEMIS: Automated Robotic Examination Intelligent System
CBE: clinical breast examination
CRUK: Cancer Research United Kingdom
HCP: health care professional
MD: mean difference
NHS: National Health Service
R-CBE: robotic clinical breast examination

## Data Availability

The data sets generated for this study can be found on GitHub [42].
