# Supplementary material for: Acceptability of Automated Robotic Clinical Breast Examination: Survey Study
Source: J Particip Med. 2023 Apr 3;15:e42704. doi: 10.2196/42704 (PMC10131668; doi:10.2196/42704)
Supplement: Multimedia Appendix 1 [file jopm_v15i1e42704_app1.docx]

**Automated Device Examination for Breast Health Monitoring**

**Project**: ARTEMIS (Advanced Robotic Breast Examination Intelligent System).

You are being asked to complete an online survey exploring your preferences as a woman on the use of a new technology for monitoring breast health. This survey is aimed at women who have, or have had, experience of breast cancer, as well as those who do not have breast cancer or symptoms of breast cancer.

**What does taking part mean?**

You will receive a set of questions to answer. This should take you around 15 minutes. No specific preparation is necessary and we would like you to reflect on your own experience.

**Why is the study important?**

We are developing an automated breast examination system which will combine medical imaging with palpation (examination through touch). The aim of the device is to offer regular monitoring for women who do not have symptoms of breast cancer. By detecting any changes which could lead to cancer earlier we have a better chance of successfully treating it.

The needs and preferences of women are at the forefront of the research. By completing this survey you will help us make sure that the technology meets the needs of women who may use it in the future.

**What is the device?**

The final device has not been fully developed yet. As the image below suggests, in a possible version of the device, the user would enter a private booth and sit comfortably at an adjustable seat in front of the automated system, leaning forward. The user would need to undress to the waist or similar, e.g. unbutton their shirt, so that their breasts are in direct skin-contact with the scanning controls. These will be soft parts which will come into contact with each breast in order to scan its entire surface. During this scan, the user would experience a gentle pressure (similar to examination by hand) from the moving sensors. The procedure would be entirely automated, with no human operator present inside the booth, but there will be a professional outside the booth to take the booking and answer possible questions.


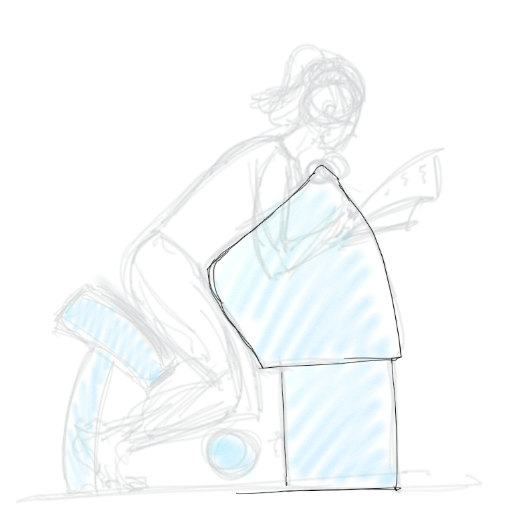


Concept drawing of a Potential Design

**Are there any benefits or risks to me in completing this survey?**

There are no known risks to you from completing this survey. While completing the survey you will be asked to imagine that you are using the technology, but you will not actually have an examination. Your responses to the questionnaire will be confidential and you will not be able to be identified as a participant in the study. After completing the questionnaire, you will have the option to enter a prize draw for a £20 Amazon voucher.

Please click here to see the full **Participant Information Sheet** [hyperlink]

Please click here to complete the **Consent Form** [hyperlink]

| Yes | No |
| --- | --- |
|  |  |

Have you ever had breast cancer?

**Specific information for device development:**

As explained in the introduction, we are working to develop an automated device that could check women’s breasts without an examination by a doctor or nurse. This would be aimed at women who don’t have symptoms. Women with symptoms would still go to their GP as they do now. It would be a way of monitoring breast health.

1. Thinking about such a device, how important is it that…

|  | Not important | Slightly important | Moderately important | Quite important | Essential | I don’t know |
| --- | --- | --- | --- | --- | --- | --- |
| You are able to adjust the speed of the device’s moving parts that touch your skin? |  |  |  |  |  |  |
| You are updated on progress at each stage of the examination? (e.g. how long is left such as a progress bar or breakdown of the current examination step shown on a screen) |  |  |  |  |  |  |
| The parts of the device that touch your skin are disposable (only used for you)? |  |  |  |  |  |  |
| You have information on the cleaning processes for the device and booth? |  |  |  |  |  |  |
| The booth where the device is located is close to your home or work? |  |  |  |  |  |  |
| Appointments are easily available at a time that suits you? |  |  |  |  |  |  |
| The device provides links to appropriate support from a health professional? |  |  |  |  |  |  |

1. Provided the booth is comfortable, how long would you be willing for the examination to last to get an accurate assessment from the device?

|  | 5 minutes | 10 minutes | 15 minutes | 20 minutes | 20+ minutes |
| --- | --- | --- | --- | --- | --- |

1. How important is it that the examination does not take longer than this?

|  | Not at all important | Slightly important | Moderately important | Quite important | Essential |
| --- | --- | --- | --- | --- | --- |

1. Would you like to be able to pause the examination to have a break? e.g. to answer your phone, or to stretch

| Yes | No | I don’t mind |
| --- | --- | --- |

1. Parts of the device will touch your skin during the examination. Would you prefer that:

| The parts are hard | The parts are soft | It doesn’t matter either way | I don’t know |  |
| --- | --- | --- | --- | --- |

1. Where would you prefer the private booth with the automated device to be located? Please rank the following options from 1 (most preferred) to 5 (least preferred)

| Pharmacy |
| --- |
| At the GP |
| At the shopping centre |
| At work |
| Other (enter free text): |

**Results and communication:**

The result of the examination by the device would be either ‘normal breast tissue’ (no further investigations needed) or ‘abnormal breast tissue’, in which case you would be advised to make an appointment to see your GP as soon as possible. If you were using this device:

1. How long would you be happy to wait at the booth if it meant your results could be available straight away (before you left the booth)?

| How long for exam | Less than a minute | 5 minutes | 15 minutes | 30 minutes | 1 hour | I wouldn’t want results before I left |
| --- | --- | --- | --- | --- | --- | --- |

1. How long might you be happy to wait to have your results delivered to you if they weren't available straight away?

| 24 hours | 72 hours | 1 week | 2 weeks | I don’t mind |
| --- | --- | --- | --- | --- |

1. The device will be able to give you a result, how would you like this to be communicated? Please rank the following options from 1 (most preferred) to 6 (least preferred)

| Written information only |
| --- |
| Written information with accompanying pictures / diagrams |
| Written and interactive information, available through a personalised app (eg a 3D graphical map of the breast) |
| A verbal summary (this would come from an audio speaker in the device and would not be able to respond to questions) |
| Other – (enter free text) |

1. What information would you like included in your results? (please tick all that apply)

| Instructions as to when you should next have your breasts checked by the service if the result is normal |
| --- |
| Detailed information of what will happen next if the result is abnormal and you require follow up with a health professional (referral process) |
| Links to where you can get emotional support |
| Information on different possible causes for any abnormal findings and breast pain |
| Other (enter free text): |

1. If the result of the examination by the device was “healthy breast tissue” how would you prefer to receive the results? Please rank the following options from 1 (most preferred) to 8 (least preferred)

|  |
| --- |
| Results provided at the time of the examination with information displayed on a screen once the assessment is complete |
| Results provided at the time of the examination with information displayed on a screen that I can print out |
| Results emailed to me once the assessment is complete |
| Results posted to me in a letter |
| Results sent to me via text message |
| Results emailed to my doctor, I want them to check the results first and then send them to me |
| I don’t need any information as long as the result is normal |
| Other – (enter free text) |

1. If the device detected abnormal breast tissue and recommended a referral to health professional, what would be your preferred option for receiving the results? Please rank the following options from 1 (most preferred) to 7 (least preferred)

|  |
| --- |
| Results provided at the time of the examination with information displayed on a screen once the assessment is complete |
| Results provided at the time of the examination with information displayed on a screen that I can print out |
| Results emailed immediately to me once the assessment is complete |
| Results posted to me in a letter |
| Results sent to me via text message |
| Results emailed to my doctor, I want them to check the results first and then send them to me |
| Other – (enter free text) |

**Overall acceptability of automated device examination for monitoring breast health:**

1. Assuming the device would be as good as an examination by a GP, would you use a free service to monitor breast health performed by an automated device?

| No, definitely not | No, probably not | Yes, probably | Yes, definitely | I don’t know |
| --- | --- | --- | --- | --- |

Please explain your answer (you can write as much or as little as you like)

1. Assuming the device would be as good as an examination by a GP, how likely are you to suggest to a friend of relative of yours that they use of a free breast health monitoring service performed by an automated device?

| 1 - Not at all likely | 2 | 3 | 4 | 5 | 6 | 7 | 8 | 9 | 10 - Extremely likely |
| --- | --- | --- | --- | --- | --- | --- | --- | --- | --- |

Why? (open text mandatory)

1. What would make you more likely to use the automated device? Please check all that apply:

| Knowing that my results would be reviewed by my GP |
| --- |
| Having more technical information about how the device works |
| Having more information about what to expect during the examination before making an appointment |
| Being able to “drop in” and have an examine without needing an appointment |
| Receiving information on how the device supports GP services |
| Receiving information on how the device supports specialist NHS services |
| Receiving a faster (direct) referral to a specialist breast clinic if your result was abnormal |
| Knowing that your data was confidential and could only be shared with your consent (e.g. with your GP) |
| Receiving information on how your data will be stored and handled. |
| Other – (enter free text) |

1. How often would you want to use a service for automated device breast health monitoring?

| Rarely or never | At least once every 12 months | At least once every 6 months | I prefer not to say |
| --- | --- | --- | --- |

1. Would you see your doctor for further investigations if the device recommended that you should?

| No, definitely not | No, probably not | Yes, probably | Yes, definitely | I don’t know |
| --- | --- | --- | --- | --- |

Open questions:

1. What other information would you need before deciding whether to use a service for breast health monitoring with an automated device examination?

As technology develops, it may become an option to use a small, hand-held automated device to monitor breast health. You would be able to use this yourself in your own home.

1. Would you prefer to use a small device in your own home compared to accessing a service like the one described above?
2. Please explain your answer (you can write as much or as little as you like):

| Worry about operating | Proficiency of Device | Convenience | Privacy | Concern for false-positives | Democratising (self-management, broader reach) | Comfort | Lack of human/professional element |
| --- | --- | --- | --- | --- | --- | --- | --- |
| 39 | 23 | 44 | 25 | 1 | 19 | 9 | 11 |

1. Is there any other information you would like to add?

**Demographics and personal preferences.**

In this section we would like to hear more about you in general. The following questions do not refer specifically to the automated device technology. We recognise that your background and experiences will be important when you make choices. We are asking these questions to better understand how preferences vary between different groups of people and to ensure that we take this into account.

1. Age Please enter your age
2. Ethnicity
3. Education level
4. Have you ever had any other type of cancer?:

| Yes | No |
| --- | --- |

1. Has a relative or close friend of yours had any type of cancer?

| Yes | No |
| --- | --- |

- 1. If Yes: What type?

1. Country of residence
2. Do you primarily access medical care via a public health system or private health care?
3. How do you think your risk of developing breast cancer compares with other women your age?

| Much lower | A little lower | About the same | A little higher | Much higher | I don’t know |
| --- | --- | --- | --- | --- | --- |

1. Do you think routine cancer screening tests for healthy people are almost always a good idea?

| Yes | No | I don’t know |
| --- | --- | --- |

1. Do you have regular breast cancer screening with mammography?

| Yes | No | Prefer not to say |
| --- | --- | --- |

1. If no, why?

| I am not yet eligible |
| --- |
| I am no longer eligible |
| I had a previous bad experience |
| I find it uncomfortable |
| I prefer not to say |
| Other – (enter free text) |

1. In general, how likely are you to use new technology when it is released?

| Not at all likely | Slightly likely | Moderately likely | Quite likely | Extremely likely |
| --- | --- | --- | --- | --- |

1. In general, what do you think of increased use of new technology in healthcare?

| Very bad idea | Bad idea | Good idea | Very good idea | I don’t know |
| --- | --- | --- | --- | --- |

1. How often do you check your breasts?

| Rarely or never | At least once every 6 months | At least once a month | At least once a week | I prefer not to say |
| --- | --- | --- | --- | --- |

1. Are you confident that you would notice a change in your breasts?

| Not at all confident | Slightly confident | Moderately confident | Quite confident | Extremely confident | I prefer not to say |
| --- | --- | --- | --- | --- | --- |

1. Would you use a free service to monitor breast health with physical examination by a trained healthcare worker?

| No, definitely not | No, probably not | Yes, probably | Yes, definitely | [blank] |
| --- | --- | --- | --- | --- |
